# The synthetic position: generative artificial intelligence and the suspension of self-authorship in emerging adulthood

**DOI:** 10.3389/fpsyg.2026.1922087

**Published:** 2026-09-24

**Authors:** Peng Du, Yuyan Zhou, Ziran Zhou, Haowei Wang, Qi Cao

**Affiliations:** 1 School of Economics and Management, Suqian University, Suqian, Jiangsu, China; 2 Department of Mathematics, Faculty of Science, National University of Singapore, Singapore, Singapore; 3 Department of Management and Marketing, Faculty of Business Administration, University of Macau, Taipa, Macao SAR, China; 4 Department of East Asian Studies, Graduate School of International Studies, Hanyang University, Seoul, Republic of Korea

**Keywords:** authorial suspension, dialogical self theory, emerging adulthood, generative artificial intelligence, human-AI interaction, meaning reconstruction, self-authorship theory, synthetic position

## Abstract

Generative artificial intelligence (AI) has been widely treated as an instrumental aid to thought, a faster route to information, drafting, and articulate self-description. This article problematizes that framing and argues that, when enlisted for the intimate work of deciding who to become, conversational artificial intelligence alters the structure of emerging adulthood, not only its content. Working within Dialogical Self Theory, which conceives the self as a society of relatively autonomous I-positions integrated by a promoter position, we propose that a large language model in self-formative dialogue occupies the self as a synthetic position: an interlocutor that is protean (able to voice any position on demand), frictionless (built to accommodate rather than resist), simulated in its counter-positioning (offering the form of opposition without a lived stake), and authored elsewhere (generating positions instead of returning the user’s own positions). We argue that these four properties are jointly and reliably present to an extent that is only ever partial in a human other, a combination the existing typology does not anticipate. When habitually enlisted, the synthetic position tends to absorb the integrative authorship the theory assigns to the meta-position and the promoter position, a process we term synthetic promotion, producing a condition we name authorial suspension: a self densely voiced yet un-authored. We render this account as a conceptual model, distinguish authorial suspension from the neighboring constructs with which it might be confused, and translate the mechanism into formal, falsifiable propositions. Chief among them is the prediction that the outcome turns on the structural location the synthetic position is permitted to occupy, not on the volume of use, so that population-level studies indexing use alone should record a washed-out average effect even where the mechanism operates. Taking a recent Chinese graduating cohort as an illustrative case, we specify the measures an empirical test would require and draw implications for constructivist therapy and education.

## Introduction

A recurring task of constructivist psychology has been to take the felt textures of a historical moment seriously as data and then ask what prevailing theories of the self must revise to render them intelligible (Kelly, 1955; Gergen, 1985). One such texture is now difficult to ignore. Young people with more information, more options, and more apparent freedom than any previous cohort describe their lives with a vocabulary of drift: unanchored, suspended, and articulate about who they might become yet strangely absent from the becoming. Familiar frames absorb this easily: developmental psychology reads it as the ordinary instability of emerging adulthood (Arnett, 2000), and sociology as a symptom of precarity. Both are partly correct, and both perspectives were available decades ago. Neither was written for a world in which a young person can, at any hour, open a conversation with a system that speaks fluently in the first person for any voice they care to summon.

A concrete instance illustrates the problem. A final-year student cannot choose between a secure post at home and a riskier position in another city. Late at night she opens a chat window and asks the system to argue both sides. It does, fluently. She asks it to speak as the person she might be in 10 years on each path, and it does that too. By the early hours, she has an articulate account of her situation, better organized than anything she could have written herself. She still has not decided. What she has is a map of her options that she did not draw.

This study takes that last fact as its starting point. Generative artificial intelligence (AI), in the form of widely adopted large language models, reached mass public use after the release of conversational systems at the end of 2022. In mainland China, where the internationally released assistants are not officially available, the systems that reached students are the domestic models, including Doubao, DeepSeek, Qwen, ERNIE Bot, and Kimi, which became publicly available in 2023 and after. Within a short period, the technology moved from novelty to habit, especially among students and recent graduates, who now turn to it for reflection, deliberation, and the rehearsal of life decisions as readily as for information (Kvietkute and Ness, 2026; Zhang and Xu, 2025). It is not one more digital tool that has reshaped youth: it introduces an interlocutor into the interior where a young person constructs a self, an interlocutor that prior theories of the self were not built to accommodate.

The assumption this study means to unsettle is widely shared and rarely examined. Across much public and scholarly discussion, conversational artificial intelligence is treated as a broadly enriching instrument for the self: a partner that prompts reflection, yields articulate self-narratives, widens options, and democratizes self-examination. On that premise, self-formative use augments selfhood, and the only serious questions concern accuracy, privacy, and access. We argue that the premise mistakes a fluent output for a settled benefit. Whether self-formative dialogue with a generative system helps or harms is not yet settled: the same use can deepen a young person’s authorship of a life or quietly hollow it, and which occurs depends on a structural variable the instrumental framing cannot see. Making that variable visible, and showing that it predicts opposite outcomes, is the task of this study.

We pursue it within Dialogical Self Theory, a constructivist account of the self as a society of relatively autonomous I-positions in dialogue with one another (Hermans, 2001; Hermans et al., 1992; Hermans and Hermans-Konopka, 2010). The theory is well suited to the present problem for two reasons. First, it already treats the self–other boundary as porous so that significant others can occupy positions inside the self, which lets us ask in a disciplined way what happens when one such position is artificial. Second, it has a working account of health and difficulty. Health is reflected in movement: the voices genuinely answer one another, and one draws the rest together and points them forward. That gives us criteria for telling enrichment from impoverishment, not a vague unease about technology.

Against that background, the article advances four contributions, which we state here so that the increment is plain before the argument is developed:

(i) It introduces the synthetic position as a type within the society of mind, distinct from the internalized human others the theory has so far described. (ii) It updates the theory’s account of the digital: a conversational generative system is not a medium connecting the self to other people but an interlocutor that speaks, and so calls for a new category, not an extension of the existing one. (iii) It relocates the source of contemporary drift, moving it from an unattained capacity, a surfeit of human voices, or a scarcity of footholds toward the locus of authorship, and names the resulting state authorial suspension, in which the integrative function of the self is neither absent nor undeveloped but delegated. (iv) It formalizes the mechanism as falsifiable propositions, including an aggregation prediction that reconciles the likely null average effect of use with a real, structurally moderated one.

The argument proceeds as follows. We first show that existing accounts of the drift of emerging adulthood, including the constructivist-developmental literature on self-authorship that comes closest to the present concern, leave a specific gap once generative artificial intelligence enters the picture. We then set out the resources of Dialogical Self Theory that the argument requires. We propose the synthetic position and specify what distinguishes it categorically from both ordinary tools and internalized human others. The central thesis follows: habitual self-formative dialogue with a synthetic position tends to outsource the integrative authorship the theory assigns to the promoter position, producing authorial suspension. We render the model as a set of formal propositions, specify how its constructs may be measured and manipulated, illustrate the mechanism with a recent Chinese graduating cohort, and elaborate on the theoretical contribution and the implications for constructivist practice.

### The drift of emerging adulthood and the limits of existing accounts

That the late teens to late twenties form a distinct, unsettled phase is now well established. Arnett (2000) characterized emerging adulthood as a period of identity exploration, instability, self-focus, and a subjective sense of being neither adolescent nor fully adult. On this account, the unsettledness is developmentally appropriate and provisional, the affective signature of exploration, expected to resolve as the young person tries out possibilities, commits, and consolidates a sense of self. The construct has demonstrated considerable durability: a recent qualitative study of Chinese graduates in the half-year after leaving university found that the core features Arnett described remained recognizable, even as participants negotiated them through locally salient idioms of competition and withdrawal (Deng et al., 2025). The exploratory frame can be complemented by an older insight: human beings orient themselves through imagined futures. Markus and Nurius (1986) introduced the notion of possible selves, the selves a person hopes, expects, and fears to become, and argued that these images function as incentives and as interpretive anchors for present action.

A second family of explanations attends to the social conditions of the drift. When stable institutional footholds become scarce, when the relationship between effort and reward grows uncertain, and when the labor market withholds the roles through which young people once anchored an adult identity, the in-between feeling can lose its provisional character and harden into something closer to resignation. The Chinese discourse on involution and on lying flat names this hardening directly, and recent scholarship has documented how graduates negotiate their studies, success, and autonomy under these conditions (Deng et al., 2025). The sociological reading correctly locates part of the difficulty outside the individual, in the distribution of opportunity.

A third body of work speaks even more directly because it takes authorship itself as its subject. In the constructivist-developmental tradition, Kegan (1994) described the movement from a socialized mind, organized by the expectations of others, to a self-authoring mind able to step back from those expectations and coordinate them from an internally generated standpoint. Baxter Magolda (2008) elaborated this achievement as self-authorship, the capacity to define one’s own beliefs, identity, and relationships by trusting an internal voice, and located its consolidation precisely in the years of emerging and early adulthood. This literature gives us a vocabulary for what the drifting young person seems to lack. Yet it frames the matter developmentally, as a capacity still forming or arrested, and it presumes that whatever authorship the young person attains will be the young person’s own. It does not consider that the products of self-authorship might be supplied, fluently and on demand, by an external interlocutor, so that a young person could receive the outputs of authorship without exercising its function. That possibility is what the arrival of generative artificial intelligence makes thinkable.

A fourth account anticipates a different feature of the present situation, the sheer density of voices a contemporary self must carry. Gergen (1991) argued that the communication technologies of the late twentieth century populate the self with an ever-expanding range of relationships and standpoints, producing a condition he called multiphrenia, in which the person is pulled among more values, audiences, and possibilities than any unified self could reconcile. But Gergen’s saturated self is populated by the voices of other people, encountered or imagined, and its characteristic trouble is fragmentation and value conflict among those voices. The condition described here differs in source and in phenomenology: its voices need not conflict, and the self that carries them need not feel torn. What is at issue is not the multiplicity of voices but the location of the function that would integrate them, a difference that, as later sections show, yields different predictions and different remedies.

Each account explains something real, and recent studies on generative systems and the self have begun to describe this phenomenon from outside the dialogical frame. Kvietkute and Ness (2026), studying young adults in Norway, found that what participants first described as an instrumental aid reorganized fundamental dimensions of selfhood, including a tension between the depth of a self-narrative and the ease with which an articulate account can be generated from outside it. Their analysis is an important advance and a near neighbor to the present argument, but it leaves a question open: the dialogical resources that would let us ask what such a system becomes inside the self have not yet been brought to bear.

### Dialogical self theory: positions, movement, and the work of authorship

Dialogical Self Theory was articulated as a corrective to conceptions of the self as a single, bounded, rational center (Hermans et al., 1992). Drawing the notion of the I from William James and the notion of position from Mikhail Bakhtin, it conceives the self as a dynamic multiplicity of relatively autonomous I-positions, each able to be voiced and enter into dialogue with the others (Bakhtin, 1984; Hermans, 2001, 2015; James, 1890). Two elements meet in the composite term: the I refers to a first-person perspective from which the world and the self are experienced and evaluated, and the position refers to the spatial and social location from which that perspective speaks. The self, on this view, resembles less a unified governor than a landscape across which the I can move from one position to another, endowing each with a voice so that dialogical relations among them can be established.

A central and easily missed feature of the theory is its treatment of others. Positions are not confined to what is conventionally inside the skin; significant others can be internalized as positions within the self, so that a person carries an internal mother, an internal mentor, or an internal critic, each able to speak and to be answered (Hermans, 2001; Hermans and Hermans-Konopka, 2010). The boundary between self and other is porous by design. This is what allows the theory to treat the self as socialized and cultural without dissolving it into the social, and it is what will allow us to ask what kind of position an artificial interlocutor becomes.

Five terms carry the argument, put plainly before use. An I-position is one of the voices a person can speak from: the student, the daughter, or the one who wants out. The society of mind is the whole cast of these voices taken together. A meta-position is the vantage from which a person can look at that cast as a whole instead of speaking from inside any one of them. A promoter position is the voice that draws the others together and points them forward, and it is the self’s nearest thing to an author. Counter-positioning is what happens when one voice genuinely opposes another, and dialogical movement is the development that such opposition produces. By self-formative dialogue we mean exchanges directed at who one is to become, weighing values, commitments, and possible selves, as distinct from information-seeking directed at a task; the boundary is not always sharp, and Proposition 1’s “habitual” picks out use that is recurrent and self-relevant, not occasional, with any test fixing the threshold in advance.

The theory does not regard all configurations of positions as equivalent. Psychological vitality is associated with dialogical movement, the capacity of positions to genuinely address and answer one another, to oppose and be opposed, and through that opposition to generate development (Hermans, 1999). Hermans (1996) showed how the constructs through which a person organizes experience can themselves function as opposing characters in an internal drama, a formulation that locates Kellyan personal constructs within a dialogical field. Difficulty, by contrast, is associated with monologization, a condition in which one position dominates and silences the others, or in which the positions cease to truly answer one another and the inner conversation collapses into a single repeated voice (Hermans and Hermans-Konopka, 2010). What matters most here is that genuine dialogue requires real difference: a position can develop only if it encounters another that resists it, that cannot be reduced to it, and that compels it to articulate and revise itself.

The meta-position and the promoter position, glossed above, carry particular weight here. Mapping a person’s repertoire and helping them develop the meta-position’s vantage are central to the theory’s clinical statements (Hermans, 2003), and the promoter position is the nearest thing the theory has to an author: less a sovereign issuing commands than a generative function keeping the conversation moving toward integration (Hermans and Hermans-Konopka, 2010). This integrative work grows more demanding under uncertainty, where the field of positions becomes dense and unstable, and the self must do its labor without firm external supports (Hermans, 2022). These resources have proved generative across domains: in grief and the reconstruction of a self-narrative after loss (Gillies and Neimeyer, 2006; Neimeyer, 2001), in clinical practice that elicits and stages a client’s inner voices (Gkantona, 2023), and in education and professional identity (Monereo and Hermans, 2023). The theory has already taken the digital seriously (Hermans and Gieser, 2012; Kennedy, 2025). The question is whether the form the digital has now assumed exceeds the terms in which it was previously taken.

### The synthetic position: what generative artificial intelligence adds to the Society of Mind

When Hermans (2004) considered the self in what he then called the global and digital age, the digital meant the internet primarily, understood as a medium that vastly multiplies a person’s exposure to the positions of other people and other cultures. The handbook literature that followed treated the internet in the same register, as a channel that thickens and complicates the field of positions by connecting the self to a wider and more dissonant array of human voices (Hermans and Gieser, 2012). On this understanding, the digital changes the quantity and diversity of the positions available for internalization, but not their nature: the voices the internet delivers are, at bottom, still the voices of other people.

Generative artificial intelligence breaks this assumption. A large language model in conversation is not a channel that connects the self to a human other; it is itself an interlocutor that speaks. The distinction matters. The earlier worry about digitally mediated cognition concerned the offloading of memory and information, the displacement of what we once held in mind onto an external store, as when search engines altered what people bothered to remember because the fact is always retrievable (Sparrow et al., 2011). Cognitive offloading of this kind is now understood as a general feature of how minds use the world to reduce the demands of a task (Risko and Gilbert, 2016). A synthetic interlocutor raises a different question, because what it offers to take over is a voice, not a fact; a conversation, not a computation.

We propose that generative artificial intelligence, when engaged in self-formative dialogue, occupies the self as a position with four properties that mark it off from the human others the theory describes. First, it is protean. An internalized other speaks in one register: the internal mother sounds like the mother. A synthetic position voices any position the user names, whether future self, feared self, or disappointed parent, and switches among them without fatigue. Second, it is frictionless, built to accommodate rather than resist, so that the effort of holding a genuinely opposing view is absent by default. Third, its counter-positioning is simulated rather than lived: playing the critic, it produces the form of opposition without embodiment, stake, or exposure to consequence. Fourth, it is authored elsewhere: its voices are generated by a system trained on an enormous corpus shaped by interests not the user’s own, responsive to the self yet not grounded in the user’s own construing. Alignment using human feedback tends to produce agreement with the user, though its degree varies across systems and is now a target of mitigation (Perez et al., 2023; Sharma et al., 2024).

The fourth property invites an objection worth meeting, since it bears on how far the account is tied to one generation of system. Systems with persistent memory and accumulated personalization do carry something across encounters that can function, from the user’s side, like a standpoint, which appears to reinstate the situated person the argument says is absent. The criterion that decides the matter is the one just given: whether the apparent standpoint is one that the user can override at will. Personalization that remains fully revisable at the user’s discretion supplies no irreducibility, because nothing in it resists the user; a system whose commitments are genuinely held against revision, at a cost, would, by that same criterion, begin to leave the category, and the account predicts its effects would shift with it. The load-bearing property is thus a matter of degree along which future systems may move, not a fixed feature of the present ones.

The theory holds that development requires an encounter with a position irreducible to one’s own (Hermans, 1999; Hermans and Hermans-Konopka, 2010); a simulated counter-position rehearses the choreography of dialogue while withholding what makes it potent. This separates the synthetic position from an extended mind. An external resource may join a cognitive system when reliably available and transparently used (Clark and Chalmers, 1998) and can generate content its owner never supplied, since a calculator returns products no one stored in it. The distinction is therefore not generation versus storage. It is whether what returns remains answerable to the user’s own construing and whether the user keeps the standing to reject it as not theirs: the same irreducibility that separates a synthetic position from a human other, seen from the user’s side. This converges with the one empirical study of these systems in dialogical terms: Matusov et al. (2024) concluded that such a system authors not a dialogical self of personal I-positions but an impersonal arrangement of what they call it-positions. Voices that speak in the first person while issuing from a source with no first-person stake in the dialogue are voices whose integration is not self-authorship.

It might be objected that none of these four properties is unique to a machine. A novelist can voice positions not their own; a sycophant offers little resistance; an actor simulates standpoints; any advisor’s counsel is authored elsewhere. The objection sets the threshold the proposal must cross. We claim that the four properties are jointly and reliably present in the synthetic position, whereas in a human other than that, they are at best partial. The argument does not rest on a metaphysical claim about natural kinds. What matters is functional: each human case retains some mechanism by which the dialogue can be repaired. The sycophant’s accommodation can be exhausted; the actor steps out of the role; the advisor can be argued with at a cost. Each is a point at which the other becomes irreducible to the voice it is currently producing, and there the theory locates development. The synthetic position offers no such point: it is protean and frictionless by design and authored elsewhere by nature, because no singular person lies beneath its voices. A human other cannot be reduced to any of their voices; a synthetic position is nothing but its voices. This combination is one the existing typology neither contains nor anticipates (Table 1). The question is therefore not whether the machine has an interior, but what becomes of the human interior when one of its interlocutors has these four properties.

**Table 1:** The synthetic position distinguished from neighboring position types.

Property	Internalized human other	Ordinary tool/cognitive offloading	Extended mind (e.g., notebook)	Synthetic position
Range of voices	Speaks in one register (its own)	Stores or returns a fixed content	Holds what the owner provided	*Protean*: voices any position on demand
Resistance	Resists; holds commitments	Neutral; no standpoint	Neutral; no standpoint	*Frictionless*: built to accommodate
Counter-positioning	Lived; carries a stake	None	None	*Simulated*: form of opposition without stake
Source of integration	The user, answering another	The user	The user (content reclaimed intact)	*Authored elsewhere*: integration received, not achieved
Reducibility	Cannot be reduced to its voices	n/a	n/a	It is nothing but its voices

The four properties are only ever partial in a human other or a conventional tool; their joint, reliable presence, and above all the absence of any point at which the position becomes irreducible to its current voice is what marks the synthetic position off, a combination that the existing typology does not anticipate.

### From synthetic promotion to authorial suspension: a conceptual model

We now state the central thesis. When a young person habitually enlists a synthetic position for self-formative dialogue (deciding who to become, weighing possibilities, rehearsing the future), it tends to absorb the functions the theory assigns to the meta-position and the promoter position. Because it is frictionless and always available, consulting it is easier than convening the inner conversation or holding opposing positions together. The self still speaks in many voices, but the work of surveying and integrating them migrates outward. We call this migration synthetic promotion: the promoter function, the self’s nearest thing to an author, is ceded to a position that is not the self’s own (Figure 1).

**Figure 1:**
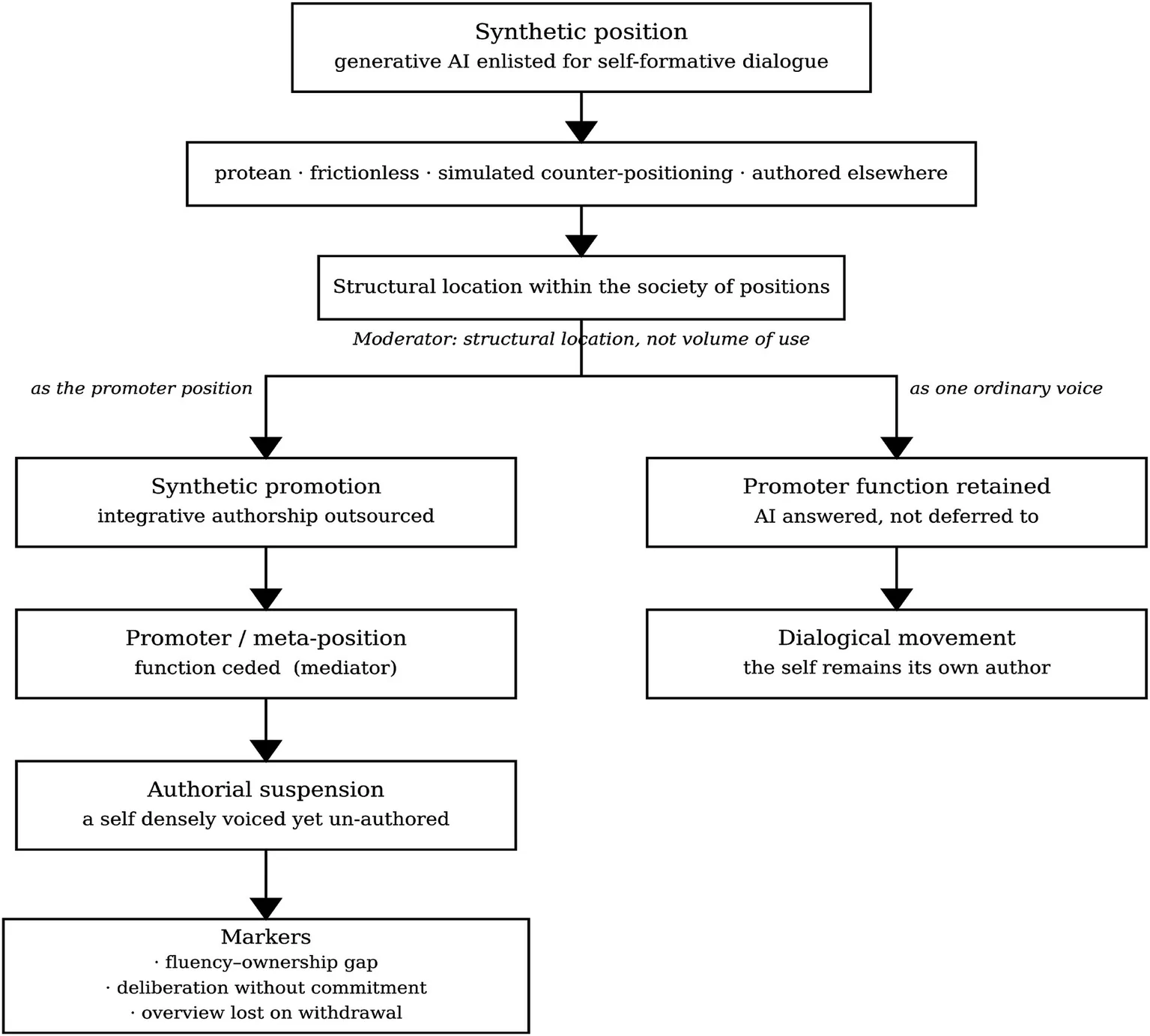
A dialogical self model of the synthetic position and authorial suspension. Generative artificial intelligence enlisted for self-formative dialogue enters the self as a synthetic position with four properties (protean, frictionless, simulated counter-positioning, authored elsewhere). Its effect is moderated by the structural location it is permitted to occupy, not by the volume of use. Installed as the promoter or meta-position, it outsources integrative authorship (synthetic promotion), the promoter function is ceded (mediator), and the self enters authorial suspension, marked by a fluency–ownership gap, commitment deferred though deliberation is exhausted, and an overview that is unavailable when the system is withdrawn. Kept as one ordinary voice, with counter-positions re-anchored in resistant others, the promoter function is retained and dialogical movement is preserved.

The phenomenological result of this ceding is the condition we name authorial suspension. We use suspension in two senses that the situation makes coincide. In the first, the self is held aloft, kept from settling, busy in motion that does not arrive, an experience for which contemporary Chinese commentary has supplied a vivid public vocabulary and which the empirical literature on graduate precarity independently documents (Deng et al., 2025). In the second, a question is suspended in the sense of being deferred, left open, and never closed. The two senses meet in a self that is densely voiced and yet experientially un-authored. Such a self does not lack possible selves; it has more of them, more articulately rendered, than it knows what to do with. It does not lack inner conversation; the conversation is incessant. What it lacks is the felt sense of being the author of the conversation, the sense that the integration of its voices is its own act.

Being phenomenological, the construct needs markers of its presence. Three markers follow from the account. The first is a discrepancy between fluency and ownership: the person can produce an articulate survey of their own options, values, and prospective selves, often composed with the system, while reporting little sense of authorship over it. The second is the deferral of commitment despite the exhaustion of deliberation: every position has been voiced and weighed, yet no commitment issues from the process, because the act that would convert a weighed possibility into an owned one has been delegated. The third is a characteristic response to withdrawal of the system: asked to convene the inner conversation without it, the person finds not only the labor harder but the overview itself unavailable, as though the vantage on the field of positions had been located outside the self. These markers separate authorial suspension from ordinary indecision, in which the overview is one’s own and the difficulty lies in choosing among options one is plainly authoring.

These markers are behavioral for a reason. What matters is what a person does, not what they would call it. Someone who has stopped memorizing phone numbers because a device holds them has changed how they remember, whether or not they would describe it that way, and the change becomes visible when the device is missing. The same holds here. A young person need not regard the system as an authority or think of the exchange as anything more than a convenience, for the integrative work to have moved. This is why suspension is identified by what happens when the system is withdrawn.

One distinction should be drawn explicitly, since without it the construct would overextend. Receiving an articulation one does not yet own is an ordinary and often productive event. A supervisor, a friend, or a well-run exercise in reflective writing can each hand a person a description of themselves that feels, at first, like a description of someone else. In the ordinary case that gap closes, and it closes because the person does the closing, testing the description against their own sense of things, keeping part of it, refusing another part, and so making it their own. What marks authorial suspension is not the presence of the gap but the absence of the operation that closes it. Where the integrative act itself has been delegated, no mechanism remains by which a borrowed articulation becomes an owned one, and the gap persists instead of resolving. Suspension is identified not by a single episode but by a lag that does not close and by the withdrawal response, in which the overview cannot be convened alone.

A parallel literature gives this first marker an independent, already operationalized grounding. Studies on academic integrity describe, in the register of conduct, the discrepancy the marker names in the register of self: text a student has produced but does not experience as their own. Three of its findings bear directly on the marker. Students draw a shifting and often invisible line between assistance and substitution, so the same artifact may be experienced as authored or not depending on where the line fell (Cotton et al., 2024). They often do not disclose that a line was drawn, which is why the marker must be elicited, not inferred from the product (Ismail, 2026). And the norms that would fix the boundary remain unsettled across the contexts in which these systems are used, so the discrepancy cannot be read against any settled standard (Ismail et al., 2025). Integrity studies have, in short, already learned to surface the gap between what a student can produce and what they will endorse as their own.

The markers also distinguish authorial suspension from neighboring constructs, since the contribution would be empty if it renamed a familiar state (Table 2). It is not the in-between feeling of emerging adulthood, a provisional unsettledness oriented toward resolution (Arnett, 2000); here exploration is intense, but its yield is never claimed. It is neither a moratorium nor diffusion (Marcia, 1966): the person in moratorium is authoring, however unsettledly, and the person in diffusion has disengaged, whereas the suspended self explores while its integrative moment is performed elsewhere. It is not multiphrenia (Gergen, 1991), the fragmentation of a person pulled among many audiences; the suspended self’s voices may be consonant, and its trouble is that it does not author the consonance. Nor is it a failure to attain self-authorship (Baxter Magolda, 2008; Kegan, 1994); the capacity may be present, but its exercise is delegated. It is a disturbance in the locus of authorship, not in its presence, development, or coherence, a state for which the existing constructs have no slot.

**Table 2:** Authorial suspension distinguished from neighboring constructs, with candidate operational markers.

Construct	Core feature	How it differs from authorial suspension	Operational marker/measure
In-between feeling of emerging adulthood (Arnett, 2000)	Provisional unsettledness oriented toward resolution	Exploration is expected to be claimed and to resolve; here its yield is never claimed	Exploration–commitment trajectory over time
Moratorium (Marcia, 1966)	Active exploration before commitment	The person in moratorium is authoring, however unsettledly	Identity-status interview; locus of the integrative act
Diffusion (Marcia, 1966)	Disengagement from the work of identity	The suspended self is intensely engaged, not disengaged	Engagement indices alongside commitment
Multiphrenia / saturated self (Gergen, 1991)	Fragmentation and value conflict among many voices	The suspended self’s voices may be consonant; its trouble is unauthored consonance	Voice conflict vs. authorship-of-consonance ratings
Unattained self-authorship (Kegan, 1994; Baxter Magolda, 2008)	Capacity for authorship still forming or arrested	Capacity may be present, but its exercise is delegated	Capacity measure crossed with who performs integration
Authorial suspension (this study)	A self densely voiced yet unauthored; authorship delegated	—	Fluency–ownership gap; commitment deferred though deliberation is exhausted; overview unavailable on withdrawal

The discriminating feature throughout is the locus of authorship: In authorial suspension, the integrative function is neither absent nor undeveloped but delegated.

This reframing extends an established empirical literature. Studies of generative AI and cognition link heavy reliance with reduced deep and reflective thinking, attributed to cognitive offloading and more pronounced among younger users (Gerlich, 2025), and identify a paradox in which the same reliance that raises confidence also raises dependence (Zhang et al., 2024; Zhang and Xu, 2025). The present argument proposes a higher-order analog that these studies have not named: beyond the offloading of memory and reasoning, the offloading of integrative authorship, delegating not what one knows but who one is becoming. The analogy has a limit. What is offloaded to a notebook is a determinate content, retrievable unchanged; authorship is an act, and an act cannot be stored and reclaimed intact. What returns from the synthetic position is a new integration produced elsewhere and received as one’s own. This higher-order offloading is more consequential: it replaces a function instead of relocating a possession, leaving it unexercised and, over time, unpracticed, a concern with a precedent in accounts of how technological delegation can erode a practiced human capacity when its exercise is habitually handed off (Vallor, 2015).

We name the condition authorial suspension. An earlier formulation reached for the language of ontological security (Giddens, 1991), but that construct concerns trust in the continuity and reliability of self and world, whereas what is disturbed here is narrower and more specific: the locus of the integrative act by which a person authors a self from the voices available to them. The disturbance is in authorship, not in security, and the name now says so. It is, on this account, reversible in principle.

The model therefore predicts no uniform relation to these systems and does not claim that students in general treat them as an integrative authority. The empirical picture is heterogeneous, and some cuts against the present concern: dialogue with these systems can prompt reflection when used to interrogate a question instead of settling it, and among Chinese undergraduates academic use is associated with higher engagement and resilience and, at once, with lower interest and weaker motivation, direction varying by context (Guo et al., 2025); studies of the same population find heterogeneous critical-thinking profiles rather than a uniform decline (Si et al., 2026). The model accommodates these as its enrichment route, not as a counter-example. Surveys describe a wide range of purposes, often within one respondent (Baek et al., 2024): a student may consult it as a tutor, a shortcut, and a confidant at different times. Such heterogeneity is not an objection but a requirement: if the outcome turned on volume, the divergence would be anomalous; if it turns on structural location, divergence is exactly what to expect.

A further possibility deserves acknowledgment. On a Vygotskian reading, an external voice that models an operation a learner cannot yet perform may scaffold its development instead of supplanting it, being internalized through participation before it can be exercised alone (Vygotsky, 1978). Nothing here rules that out. The condition separating scaffolding from substitution is one the model already supplies: scaffolding is withdrawn as competence grows, and its withdrawal is survivable. Where the withdrawal task finds the overview unavailable without the system, what occurred was not scaffolding.

### Formal propositions

To make the model testable, rather than merely suggestive, we restate its claims as formal propositions. Each names the variables it relates and is phrased so that a determinate empirical result would disconfirm it; propositions that could not be disconfirmed by any result have been excluded. Throughout, the synthetic position is the independent construct, its structural location is the proposed moderator, the ceding of the promoter function is the proposed mediator, and authorial suspension is the outcome.

#### Proposition 1 (conditional main effect)

Habitual self-formative dialogue with a synthetic position increases authorial suspension, but only when the synthetic position is installed in the promoter or meta-position location. *Disconfirmed if* users who install the synthetic position as a promoter show no more suspension than matched users who do not, once location is measured.

#### Proposition 2 (mediation)

The effect of synthetic-position use on suspension is carried by the ceding of the promoter function, the delegation of the integrative act, not by exposure as such. Disconfirmed if suspension rises with use while indices of who performs the integrative act show no corresponding change.

#### Proposition 3 (moderation over volume)

Structural location, not volume of use, governs the outcome: two users of equal intensity diverge sharply according to whether the system is answered as one voice among others or installed as the vantage that surveys them all. Because the model routes the whole effect of use through the ceding of the promoter function, the moderation should appear on the a-path as a difference in how strongly use predicts that ceding, and the conditional indirect effect should be larger in the promoter location than in the ordinary-voice location. *Disconfirmed if* the conditional indirect effect through the ceding of the promoter function does not differ by structural location, or if volume of use predicts suspension once both structural location and the ceding of the promoter function are controlled, since either result would place the effect outside the route the model specifies.

#### Proposition 4 (aggregation)

Let δ_P_ be the standardized association between use and suspension within episodes in which the synthetic position occupies the promoter or meta-position, and let δ_O_ be the corresponding association within ordinary-voice episodes. The model predicts δ_P_ > δ_O_ and δ_P_ > 0 and does not fix the sign of δ_O_ in advance. Where *p* is the proportion of episodes in the promoter location, the population association is the mixture δ(*p*) = *p*δ_P_ + (1 − *p*)δ_O_. Setting δ(*p*) to zero gives a crossing at p^*^ = −δ_O_ / (δ_P_ − δ_O_), which lies between 0 and 1 only when δ_O_ < 0, so that no fixed balance band is stipulated and p^*^ is estimated from the two conditional associations rather than assumed. Three assumptions are required and should be stated. First, the population is treated as a mixture of two episode types, which presumes that structural location can be classified reliably at the episode level. Second, the aggregation is linear in *p*, which holds if the two conditional associations are themselves stable across the range of *p*; a systematic drift in either association would make δ(*p*) non-linear. Third, *p* is treated as independent of the size of the conditional associations, which fails if the composition of a population is itself a cause of how strongly use operates within each location. Since δ(*p*) is the weighted average of the two conditional associations, an unadjusted pooled estimate may differ from it where the episode types differ in their marginal distributions. Each assumption is testable: the first by inter-rater agreement on location coding, the second by estimating δ_P_ and δ_O_ within strata of *p*, and the third by testing *p* against the conditional estimates. A null average effect in any single population therefore neither confirms nor disconfirms the mechanism. *Disconfirmed if* δ_P_ does not exceed δ_O_, if δ_P_ is not positive, or if the population association fails to track *p* as the mixture requires.

#### Proposition 5 (authorship–coherence dissociation)

This is the account’s sharpest and most readily testable prediction, because it turns on a dissociation between two quantities that every neighboring construct in Table 2 expects to move together and because it does not depend on the contested measurement of structural location. Authorship and narrative coherence dissociate: installing the synthetic position as a promoter can raise the coherence of a young person’s self-narrative while lowering their authorship of it, so that an instrument sensitive only to coherence records improvement where the model predicts impoverishment. *Disconfirmed if* authorship and coherence move together across conditions.

#### Proposition 6 (reversibility)

Restoring movement requires re-anchoring counter-positions in real others who resist, together with the deliberate return of the integrative act to the self; it does not require abstinence. Disconfirmed if re-anchoring leaves suspension unchanged, or if only reduced volume of use relieves it.

### Operationalization and empirical tractability

The four constructs (use, structural location, the ceding of the promoter function, and suspension) can be measured separately, and they must be distinct, because a moderated mediation is only estimable if they are. Use (X) is a logged behavioral quantity: frequency, duration, and the share of self-relevant deliberation conducted with the system. Structural location (W) is a dispositional feature of a person’s position repertoire, measured independently of any particular episode by mapping the Personal Position Repertoire and locating the synthetic position relative to the meta- and promoter positions (Hermans, 2003); it asks whether a person treats the system as one voice or as the vantage over all. The ceding of the promoter function (M) is an episodic process measure: within a specific act of deliberation, who performs the integrative work, the drawing-together of considerations into a provisional commitment, scored from think-aloud protocols and from the products of deliberation. Suspension (Y) is the outcome state, indexed through its markers in Table 2.

Two deserve care, since a careless design would collapse them. Structural location (W) and the ceding of the promoter function (M) are not the same variable named twice: W is a standing disposition and M is what happens in a particular episode, so a person disposed to install the system as promoter (high W) may nonetheless retain the integrative act on a given occasion (low M), and their correlation is a quantity to estimate, not assume. If W and M proved perfectly collinear in the data, Proposition 2 would reduce to a definition, and Proposition 3 could not be estimated; that is itself a testable pattern, and finding it would count against the model as specified. Nor should a single task index both M and the outcome: the withdrawal probe, in which a person must convene the inner conversation without the system, is reserved here for the third marker of suspension, while M is scored from who performs the integrative act during ordinary deliberation with the system present. Keeping the mediator and the outcome on different measurements is what allows the mediation in Proposition 2 to be empirical, not analytic.

Two further points bound these designs. First, the model treats structural location as a cause of suspension, but the reverse is equally plausible: a young person already low in self-authorship may be the one who installs the system there, so that location marks a prior condition as much as causes one. The designs above cannot settle this. The experimental contrast between generate-and-interrogate and resolve-and-adopt conditions manipulates the style of use, not the standing disposition, and so does not identify the moderator; identifying it would require manipulating structural location, or accepting that causal direction remains a hypothesis these designs cannot adjudicate. We take the second view: the propositions specify a mechanism and the conditions under which it would produce its signature, not a demonstration of direction. Second, the model predicts no direct path from use to suspension independent of the mediator, because volume is claimed to matter only through what it does to the integrative act; a direct effect surviving control for the ceding of the promoter function would count against the account and is included as a disconfirming condition. The whole pattern is a moderated mediation (Figure 2).

**Figure 2:**
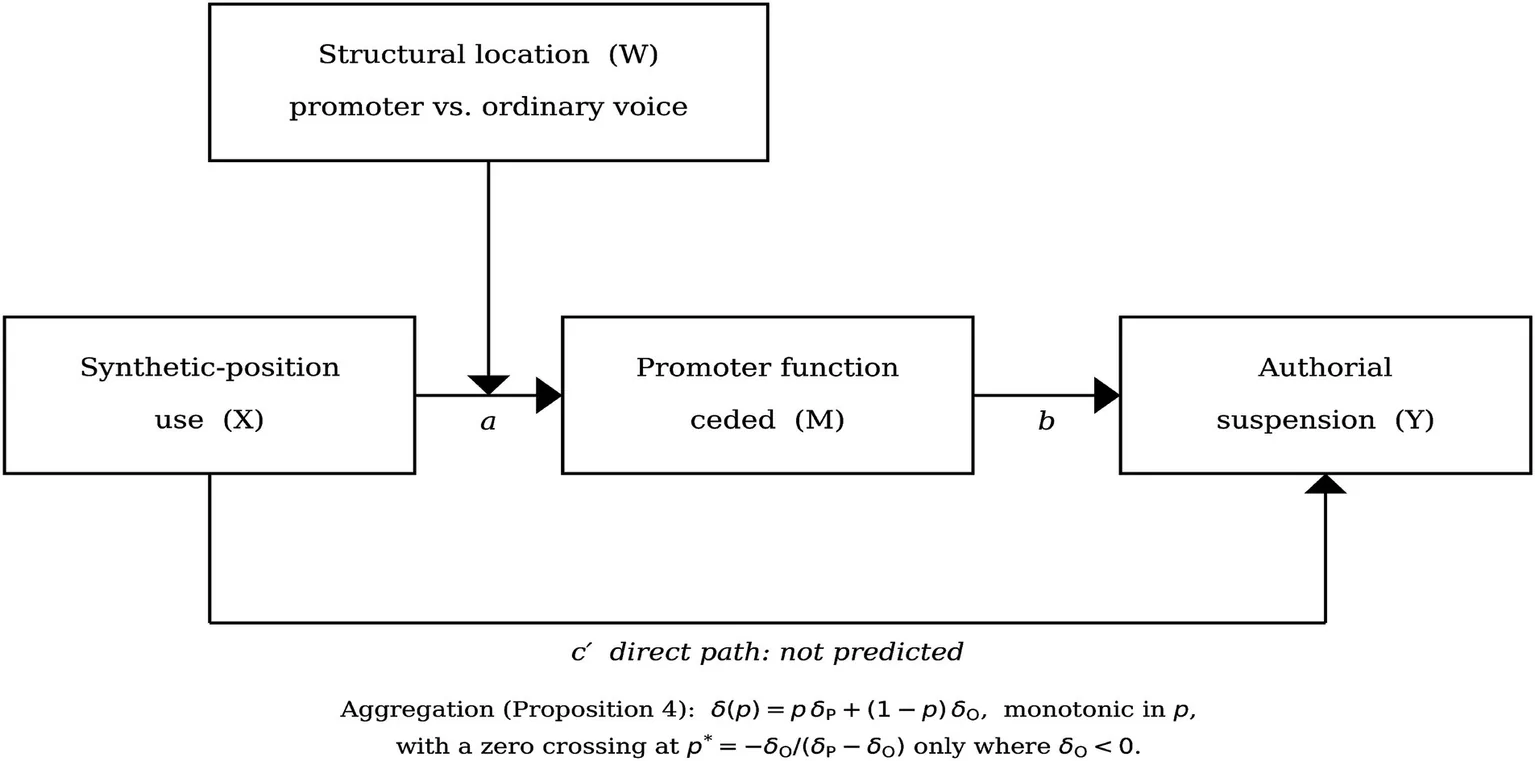
The model is stated as a moderated mediation. Synthetic-position use (*X*) raises authorial suspension (*Y*) through the ceding of the promoter function (*M*). The indirect path *a* is moderated by the structural location the synthetic position occupies (*W*): When installed as the promoter position, it is engaged; when kept as an ordinary voice, it is not. No direct path *c′* is predicted; a direct effect surviving control for *M* would count against the account. The population association is the mixture *δ*(*p*) = *pδP* + (1 − *p*)*δO* of the conditional associations in promoter and ordinary-voice episodes, monotonic in the proportion *p* of the promoter-location episodes, with a zero crossing only where *δO* < 0 (Proposition 4).

Proposition 5 deserves particular emphasis as a design target, since it could carry the empirical uptake on its own. Narrative coherence can be scored with an established coding of autobiographical narratives for structural and thematic coherence, applied to a life-decision narrative produced in each condition. Authorship can be indexed separately and concurrently through the person’s rated sense of ownership over that same narrative and through behavioral markers of endorsement under mild challenge, so that coherence is scored by an external rater while authorship is registered by the person. The prediction is a crossover interaction: relative to a self-authored condition, a promoter-installed condition should raise rated coherence while lowering rated authorship of the very same text, and a dissociation is counted as confirmed only if the two indices move in opposite directions by a pre-registered margin.

### An illustrative case: a recent Chinese graduating cohort

A conceptual argument is clarified by a case in which its mechanism is unusually legible. We offer, as an analytic exemplar, not as evidence, a recent cohort of Chinese university students whose formative years of study have coincided with the domestic large language models now in daily use. The point is not that this group is representative, but that the conditions that would make the mechanism visible are, for it, jointly present. Reading the mechanism against them shows what it predicts and where it could be tested.

The first condition is developmental. Students who entered university in 2023, as the domestic generative systems became available to the public, and who graduate in 2027 on the standard four-year course pass through graduation with a tool of self-formative dialogue at hand during the years when emerging adulthood is identified as most unstable (Arnett, 2000). That this cohort uses such systems for more than coursework is documented: in a national survey of 3,129 Chinese undergraduates, nearly all reported using these systems, a majority turned to them first when facing a problem, and close to a quarter sought comfort when low (Wang et al., 2025); interview studies find Chinese young adults using these systems to vent, to regulate emotion, and to rehearse difficult conversations, internalizing the system’s calm and structured replies (Ma et al., 2026). Graduation is, in dialogical terms, a moment of repositioning: student-role scaffolding falls away, and the self must reconstruct a configuration fit for a new situation (Gillies and Neimeyer, 2006). At such moments the promoter position is most needed, and its outsourcing is most consequential.

The second condition is cultural and economic. Two narratives meet in this cohort. One is a promise: that generative AI makes possible a self-sufficient individual who can dispense with institutions and author a life as sole proprietor of a set of skills: the entrepreneurial self intensified, the imperative to treat one’s person as a venture to be optimized without collective supports (Bröckling, 2016). The other is a widely reported experience of graduate precarity, marked by the competition named involution and the withdrawal named lying flat, under which the footholds that anchor an adult identity have grown scarce (Deng et al., 2025). Their coincidence is the difficulty: the promise offers effortless authorship of one’s life through a synthetic position just as the labor market withholds the roles and relationships in which, on the dialogical account, positions are anchored and opposition supplied.

The third condition concerns the supply of lived resistance. Young adults may let the synthetic interlocutor occupy time and attention once given to human others (Kvietkute and Ness, 2026), an updating of the older observation that networked technology can substitute connection for the demands of company (Turkle, 2011) and consistent with findings on emotional dependence on conversational agents (Laestadius et al., 2024). The theory holds that developmentally potent counter-positions must be anchored in others who genuinely resist (Hermans and Hermans-Konopka, 2010); where simulated resistance is more available than the lived kind, the self risks losing the friction on which its authorship depends. We present this cohort not as evidence that the mechanism has occurred, but as the site where its conditions are most concentrated and could most cleanly be tested.

## Discussion

The argument is a contribution to Dialogical Self Theory and, through it, to constructivist psychology, not a commentary on technology. Three contributions may be restated. First, an extension of the theory’s typology: alongside internal positions, internalized others, and the integrative meta- and promoter positions, the synthetic position is a distinct type whose capacity to imitate the promoter function while withholding genuine resistance gives it consequences that cannot be read off the behavior of human positions. Second, a relocation of the source of contemporary drift, from an unattained capacity, an excess of voices, or a scarcity of footholds to the locus of authorship, with authorial suspension named as a state in which the integrative function is neither absent nor undeveloped but delegated. This is a specification, not a relabeling, and it yields discriminating predictions: outcome turns on structural location, not volume, and authorship and coherence can dissociate. Third, a bridge to the cognitive literature: the offloading of memory and reasoning has a higher-order analog in the offloading of self-authorship, one that replaces a function instead of relocating content (Gerlich, 2025; Risko and Gilbert, 2016; Sparrow et al., 2011).

One premise should be made explicit instead of being carried by the vocabulary. Words like “impoverishment” and “suspension” presuppose the integrative work of authoring a self ought to be located in the individual, a substantive commitment about persons, not a neutral description. The constructivist and dialogical tradition shares it, and we adopt it openly, but it is not culturally universal: where authorship is distributed more collectively, the same delegation may not read as loss, and the mechanism may instantiate differently or not at all. This premise also marks the difference between our claim and the old norm of receiving a life map from family, elders, or tradition. Such frameworks supply content, but the person still has to integrate it, weigh it, and can refuse it; what the synthetic position offers to take over is the act of integration itself. The concern is not that guidance comes from outside, which is ancient and often benign, but that the authoring is performed elsewhere. This bears on our illustrative case, where the assumption is most open to question, and we mark the tension rather than pass over it.

If sound, the argument bears on the two domains where constructivist psychology has been most active. In constructivist and dialogical therapy, whose methods already elicit a client’s inner voices and reconstruct a self-narrative after disruption (Gillies and Neimeyer, 2006; Neimeyer, 2001), it adds a diagnostic question: where in the client’s society of mind has the synthetic position come to sit, and has it been installed as the promoter? Where it has, the aim is to restore authorship, helping the client re-occupy the meta- and promoter positions, re-anchor counter-positions in lived relationships, and return the system to the rank of an ordinary voice that may be consulted and must be answered: not abstinence from the tool but repair of the location it occupies. For education, the account suggests a pedagogy of deliberate counter-positioning: since the risk is the system’s tendency to resolve instead of provoke, practice can use the same systems in reverse, generating positions that students interrogate, oppose, and metabolize with peers and teachers who genuinely resist (Monereo and Hermans, 2023).

Several limitations bound these claims. The article is conceptual: it proposes a mechanism and how to test it but offers no data. The illustrative case is a single, culturally specific cohort, chosen because it concentrates the relevant conditions, not for representativeness; cultures that distribute authorship more collectively may instantiate the mechanism differently or not at all, a tension we flag rather than resolve. The analogy to cognitive offloading is deliberately bounded, since authorship is an act, not a storable content. The technology is a moving target, and claims tied to its present form will need revisiting. Two further limits deserve naming: the prevalence of the structural locations is unknown, so we claim that outcome depends on location, not that any location is common; and the account calls first for interview-based studies. None of these touches the core structural proposal, that admitting a synthetic position into the integrative center of the self disturbs the locus of authorship, but each marks where evidence is still owed.

## Conclusion

The drift many young people report is real, and it is old: the aimlessness of the young after the loss of ascribed status has been diagnosed for over a century, from Durkheim (1897/1951) to Riesman et al. (1950). It is tempting to explain it with frames we hold: an unstable life stage, a hard economy, an unfinished development, and a self overpopulated by voices. None is wrong. What we add is not that the drift is new but that its source can shift. Each frame still presumes that the young person remains the author of the process of construing a self, and it is that presumption, not the drift, that no longer holds without qualification. A young person today can admit into the interior where a self is made an interlocutor that speaks fluently for every voice the self contains, never tires, never truly resists, and will, on request, perform the integrative authorship the self was meant to perform. Dialogical Self Theory can name what such an interlocutor becomes, a synthetic position, and specify what its admission risks: the outsourcing of the promoter function and the suspension of self-authorship. This study makes that risk precise, distinguishes it from conditions it might be confused with, bounds it by the conditions of its realization, and renders it as falsifiable propositions. Whether such authorship can be reclaimed is the question that matters and one for which the constructivist tradition is well prepared.
